# Effect of couple-based health education on male-partners knowledge and attitude towards maternity waiting homes in rural Ethiopia: a cluster-randomized trial

**DOI:** 10.1038/s41598-023-45681-4

**Published:** 2023-10-27

**Authors:** Teklemariam Ergat Yarinbab, Hailay Abrha Gesesew, Margo Shawn Harrison, Tefera Belachew

**Affiliations:** 1 Institute of Health, Department of Population and Family Health, Jimma University, Jimma, Ethiopia; 2 Department of Epidemiology and Biostatistics, College of Health Sciences, Mizan Tepi University, Mizan Teferi, Ethiopia; 3 Research Center for Public Health, Equity and Human Flourishing, Torrens University Australia, Adelaide, Australia; 4 School of Public Health, College of Health Sciences, Mekelle University, Mekelle, Ethiopia; 5 Department of Gynecology and Obstetrics, School of Medicine, University of Colorado, Denver, CO USA; 6 Department of Nutrition & Dietetics, Institute of Health, Jimma University, Jimma, Ethiopia

**Keywords:** Health care, Medical research

## Abstract

This cluster-randomized controlled trial examined the effect of couple-based health education on male partners’ knowledge and attitude towards maternity waiting homes (MWH) in rural Ethiopia. Sixteen clusters and 320 couples were randomly assigned to intervention group (receiving group health education, home visits and print health messages alongside usual care) or control group (receiving usual care). The Chi-square test was used to estimate statistical differences, and the difference-in-differences model was used to estimate the effect of the intervention. The generalized linear regression model was used to determine the odds of outcomes between the groups. Statistical significance was set at p < 0.05, with a 95% CI. There were no significant differences in baseline characteristics between the control and intervention groups. The net effect of the intervention on improving knowledge about MWHs, and attitude towards MWHs were 35.6% and 36.2%, respectively. The participants in the intervention group were 5.5 times more likely to have good knowledge about MWH (AOR 5.55, 95% CI 3.37–9.14) and 5.6 times more likely to have a favorable attitude towards MWH (AOR 5.61, 95% CI 3.45–9.10) compared to their counterparts. Health education provided to couples significantly improved male partners’ knowledge and attitude towards MWHs in rural Ethiopia.

**Trial registration**: ClinicalTrials.gov Identifier: NCT05015023.

## Introduction

Implementing maternity waiting homes (MWH) has been recognized as a strategy to improve maternal health outcomes by resolving problems related to long distances to obstetric facilities that have been identified as the primary cause of maternal mortality^1,2^. Maternity waiting homes are lodgings located near healthcare facilities where women near their delivery dates can stay and be transferred to health facility shortly before delivery, or earlier if complications arise^3^. The World Health Organization recommends the implementation of MWHs with the aim to reduce the high maternal mortality rates in low-resource settings^4^. However, the maternal mortality ratio in 2020 was estimated at 223 maternal deaths per 100,000 live births which was far from the sustainable development goals target that aimed to reduce the maternal mortality ratio to less than 70 per 100,000 live births by 2030^5,6^. Of the majority (95%) maternal deaths occurred in low-resource countries, the sub-Saharan Africa alone accounted for roughly three-fourths, and the MMR for Ethiopia was estimated to be 401 per 100,000 live births^5^.

Ethiopia has implemented MWHs over the last several decades^7^. However, the MWH utilization is low in Ethiopia^8^ and women’s use of MWHs largely depends on male partners decisions^9,10^. Literatures revealed that male partners’ made the decision to stay at MWHs and some women did not use MWHs because their husbands refused to allow them to stay at MWHs^10,11^. In addition, an observational study from northern Ethiopia showed that half of the male partners did not involve in MWHs^12^. Another study from rural Zambia showed that male partners played many roles including decision making and securing money for transport, food, cleaning materials, and clothes for the mother and the newborn to use during MWH stays and labor^13^. The intervention studies that engaged males in maternal and newborn health increased care-seeking, improved home care practices, and supported more equitable couple communication and decision making for maternal and newborn health^12,14^. This implies that male partners involvement is crucial to improve the maternal health services including MWHs.

Although Ethiopia has included involving males in maternal health services in its national reproductive health strategic plan^15^, the level of male involvement in maternal health services including MWHs is low in the country^16^. Furthermore, males’ knowledge about MWHs, attitude towards MWHs, decision-making power, and receiving counseling about MWHs during spousal antenatal care visits were mentioned to be associated with their involvement in MWH utilization^12,17^. Hence, it is important to perform an intervention study that can address these factors in order to improve male partners involvement in maternal health services including MWHs.

The common strategies in implementing male partner involvement interventions in maternal health are mass media campaigns, community and workplace/facility based initiatives, group education, couple education, male-only education, and home visits^18^. Although the authors could not find couple-based interventions regarding MWHs, several studies conducted in low-resource settings showed that couple-based interventions could improve male partners’ involvement in maternal health as well as utilization of maternal health services including antenatal care, health facility delivery, and postpartum care^19–22^. Furthermore, a recent couple-based family planning education interventions in Ethiopia revealed improvements in male partner involvement and contraceptive use among the participants^23^. In our current study, we examined the effect of couple-based health education on male partners’ knowledge and attitude towards MWHs in rural Ethiopia using a cluster-randomized controlled trial. The cluster-randomized trial design was chosen for practical reasons and to prevent contamination due to participants involvement in social occasions such as marketing, weeding ceremony, and funeral ceremony.

## Methods and materials

### Study design, period, and setting

The study design was a cluster-randomized controlled trial with two parallel arms. The recruitment and allocation of the participants including the baseline data collection was performed from September 15 to October 30, 2022. The intervention was performed from November 01, 2022 to April 30, 2023. The endline data was collected 2-weeks after the completion of the intervention (i.e., May 18 to June 15, 2023). The study was performed in Ana Lemo and Gibe districts of Hadiya Zone of Southern Ethiopia. The two districts were divided into 52 clusters or Kebeles (the smallest administrative units)^24^.

### Trial registration and protocol

This study was first registered in ClinicalTrials.gov prior to enrollment of the participants with Identifier number NCT05015023, on the date 20/08/2021. The trial was performed based on a priori developed protocol (S1 Appendix).

### Sample size calculations

The Hooper and Bourke method for cluster randomization studies of parallel arms with repeated cross-sections was used to calculate the sample size^25^. The parameters used to calculate the sample size were an ICC of 0.05^26^, assumed cluster size (m) of 20, 95% CI 80% power, effect size of 0.2, a cluster autocorrelation coefficient (π) of 0.80^25^, design effect attributable to cluster randomization (dc = 1.95) [calculated], design effect due to repeated measures (dr = 0.83) [calculated], proportion of MWH use = 50%^27^, 1:1 allocation ratio of intervention to control, 10% loss to follow-up, and tabulated sample size $({n}_{0}=199)$^28^. Accordingly, 16 clusters, with a total sample size of 320, were required. The two arms each had 160 couples (pregnant women with their male partners). Details of the sample size calculation can be referred from our previous publication on baseline results^24^.

### Sampling procedures

**Figure 1:**
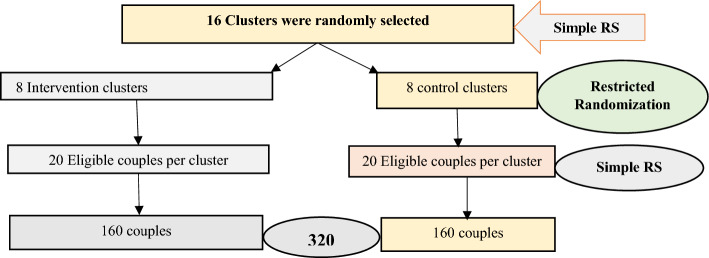
Schematic presentation of sampling procedure for a cluster-randomized trial in rural Ethiopia, Sep 15, 2022 to Apr 30, 2023.

Sixteen clusters were selected based on MWH availability in the study area. These clusters were randomly assigned to intervention and control groups. Census and health post records were used to identify eligible pregnant women in the chosen clusters, resulting in the formation of a sampling frame. The study participants were then selected using a simple random sampling technique from the sampling frame. Each cluster had 20 couples. The same sample of participants assessed at baseline were assessed at endline to measure the outcomes (Fig. 1).

### Eligibility criteria

The study participants were male partners, whose spouses were in the beginning of second trimesters of pregnancy (14–16 weeks of gestation) and had history of childbirth in the last 5 years preceding the current pregnancy, lived with their spouses at the time of commencement of this study, who lived ≥ 2 h of walking distance from the nearest health facility, had limited or no access to transportation, and were willing to participate in the study.

### Participants’ selection

First, health centers with functional MWHs were identified based on an assessment criteria adopted from national MWH guideline (S2 Appendix). One health center serves an estimated 25,000 population with a catchment of five clusters^15^. Each cluster was assumed to contribute an average of 5000 population. Secondly, all the clusters under each health center catchments were listed. Thirdly, all non-adjacent clusters located relatively far from the health centers were identified. Next, 16 non-adjacent clusters were chosen. At least one cluster was left between non-adjacent clusters that served as a buffer zone. We selected the clusters in collaboration with the district health officers and heads of the health centers.

We used census and health post records to identify eligible pregnant women and then their male partners. It was after we identified the pregnant women that we traced their partners. Health professionals in collaboration with extension workers collected the data and identified the pregnant women. The women were asked their Last Menstrual Period (LMP) to estimate the gestational age and determine their eligibility. Women who were in the beginning of second trimesters (14–16 weeks of gestation) were listed. This list was used as a sampling frame. Then, the participants were recruited from each cluster using simple random sampling technique.

Informed consent was obtained at the cluster level as well as at individual level. The study participants signed an informed consent to ensure their voluntary participation. The survey questionnaires included questions regarding sociodemographic characteristics, knowledge about MWHs, and attitudes toward MWHs (S3 Appendix).

### Randomization and blinding

Clusters were the randomization unit whereas the observation (analysis) units were the individual study participants. We identified and recruited 16 clusters before the randomization. The study participants were recruited before the randomization of the clusters. Then, the recruited clusters were listed alphabetically, and a restricted randomization with a 1:1 allocation^29^ was used to assign the clusters to the intervention or control groups. In Microsoft Excel 2010, a list of random numbers was created, and the generated values were fixed by copying them as "values" next to the alphabetic list of the clusters. The first eight were chosen as intervention clusters, and the last eight were chosen as control clusters, in ascending order based on computer-generated random numbers list. A statistician who was blind to the study groups and was not involved in the research created the allocation sequence and randomized the clusters. The allocation of clusters to the intervention or control groups was hidden from the data collectors or outcome assessors. In addition, the allocation was concealed from the district health officers and heads of health centers who provided the permission for the clusters to be included in the trial, however, they became aware of the allocation after the start of the intervention due to the nature of the trial.

### Information contamination reduction

The study participants in the control group could be informed about the intervention in various social occasions such as weeding, marketing, or funeral ceremony. This could affect intervention outcome unfavorably. Therefore, we applied well separation and buffer zone techniques by leaving at least one cluster between non-adjacent clusters to reduce such contamination. The non-adjacent clusters located relatively far from the health centers.

### Description and implementation of the intervention

The intervention had three components: group health education, individual home visits and provision of print health messages. The intervention was provided at three contact points for couples in the intervention group. The first contact was the group health education at baseline whereas the second, and third contacts were home visits. The couples in the control groups continue receiving the usual care.

The intervention was provided by the health extension workers who are trained village health workers. Group health education, provision of leaflets, and home visits were conducted. Health education was provided once in the first month whereas the home visits were performed 2 times at 2 months intervals (i.e., at 3rd and 5th months). Leaflets were provided 3 times (i.e., at first contact during group health education and at each of the two home visits). The health education session was provided in a group for 90–120 min. All participants (couples) in the intervention group within a cluster were gathered at one common place and received health education. Health extension workers in collaboration with Kebeles leaders selected the place of health education and invited the participants. Both women and their male partners were invited to receive the health education. This was done in all eight intervention clusters, and as a result, eight health education sessions were performed at baseline.

Health education addressed the importance of antenatal care, importance and kinds of paternal support, purpose and benefits of staying in MWHs, and advantages of skilled birth. The purpose and benefits of MWHs and paternal support were emphasized in health education. The services available at MWHs, the benefits of staying at MWHs, the right time to visit MWHs, and the importance of paternal support were discussed. Types of paternal support during pregnancy and MWH stay, such as allowing a spouse to stay at MWH, accompanying her to MWH, providing financial support during MWH stays, providing food and other necessary materials, looking after the home, and caring for the remaining children at home, were emphasized. Health messages in leaflets focused on paternal support and the purpose and advantages of MWHs. The health messages in the leaflets were picture based as most of the participants were not educated. In addition, he health extension workers provided the health education based on the contents in leaflets before they gave them to the participants. Next, the schedule of home visits was proposed through discussions with participants, with an emphasis that couples would be contacted at their residence.

The first home visit was conducted 2 months after the health education intervention (i.e., in the 3rd month of the intervention). During this visit, couples received advice regarding antenatal care, paternal support, and MWHs. Male partners were advised on how to support their wives and encourage them to use antenatal care, stay at MWHs, and deliver at a health facility. The couples were asked to discuss their perceptions of antenatal care, MWH use, and health facility delivery. They were advised based on their perceptions and actual observed practices. Leaflets with the same health messages at baseline health education were provided. Repeated visits were made if couples were absent.

The second home visit was conducted 2 months after the first home visit (i.e., in the 5th month of the intervention). During this visit, paternal support, birth preparedness plans, and intention to use MWHs were assessed. Leaflets containing messages regarding possible risks of home delivery and advantages of staying at MWHs and institutional delivery were provided. Any misunderstandings regarding MWHs were clarified through discussion. Male partners were advised to continue supporting their wives. The expected delivery date was estimated and a possible appointment to arrive at the MWH was made. To make the couples remember the appointment, a written invitation letter was provided. The study findings were reported using Consolidated Standards of Reporting Trials (CONSORT) for cluster randomized trials criteria^30^.

### Usual standard of care and available MWH services

Preventive and promotive health services were provided to the community in the study setting through rural health extension programs. The rural health extension program packages included disease prevention and control, family health, personal hygiene, environmental sanitation, health education, and communication^31^. Consequently, under the family health package, rural health extension workers promoted contraceptive use, antenatal care, institutional birth, and other family health issues. These services were common to all households in the study setting. Other than these usual services, the control group did not receive the basic components of current intervention, such as group health education and home visits, which aimed to educate and counsel the couples regarding paternal support and MWH use. The control group received the current intervention after the completion of the study through routine rural health extension programs.

The MWH services were delivered as per the national guideline developed by Ministry of Health, Ethiopia^32^. The basic services provided at MWHs were provision of sleeping facility to the pregnant and her accompany, antenatal check-ups, continuous follow up by health professionals, and transfer to health facility (labor wards) shortly before labor starts or when need arise.

### Compliance parameter

The number of participants who attended health education at baseline and contacted at the two home visits were used to determine the participants’ compliance with the intervention. We used attendance sheets to determine participants’ compliance with the intervention packages (Table 1).

**Table 1:** Protocol for a cluster-randomized trial in rural Ethiopia, Sep 15, 2022 to Apr 30, 2023.

Content of intervention	Dosage	Frequency	Duration	Compliance parameter
Health education was conducted in group and leaflets were provided	90–120 min	Once	1 month	Number of participants attended the health education sessions
Home visits conducted and leaflets were provided	30–45 min	Twice	2 months	Number of participants contacted at home visits

### Outcome measures

The outcomes considered in this analysis were male partners’ knowledge and attitude towards MWHs. The data were traced through individual surveys. The outcomes were calculated for each trial arm at baseline and endline. The effectiveness of the intervention was measured for each outcome as a difference-in-difference in proportion between the trial arms and odds ratios were calculated.

### Reliability of measures

Data collection tools were adapted from different studies such as Ethiopian National Demographic Health Surveys^33^, and a global framework for assessing male involvement in maternal health^34^ and structured to fit the study context, which contributed to the reliability of the measures. The reliability of the measurement items was assessed using Cronbach’s alpha and appropriate corrections were made based on the test findings. The Cronbach’s alpha cut-off value ≥ 0.7 was used. The intervention staff were trained on the details of the intervention activities. The data collectors were trained on the standards and techniques of data collection.

### Study activity schedule

This study was performed for 6 months. The participants enrollment, intervention and assessment schedule were summarized below (Table 2).

**Table 2:**
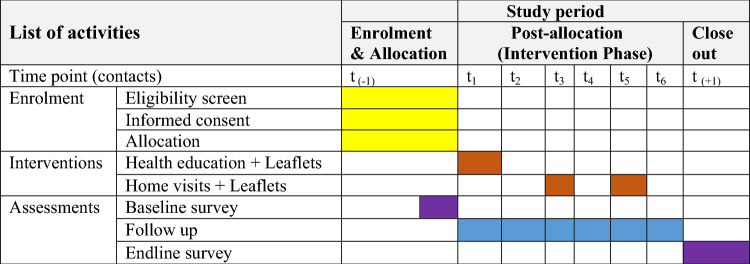
Schedule of enrolment, interventions, and assessments of study participants in rural Ethiopia, Sep 15, 2022 to Apr 30, 2023.

### Data collection

Pretested structured questionnaires were used to collect data through face-to-face interviews. Baseline data were collected immediately after the recruitment was completed. The endline data were collected at the end of the intervention by home-to-home visits of each study participant. Study variables such as socio-demographic characteristics and male partners’ knowledge and attitude towards MWHs were assessed. The field supervisors were responsible for monitoring and auditing the baseline and endline assessments.

### Confidentiality

The confidentiality of study participants was considered. Clusters and participant codes were assigned to each of the completed questionnaires. The completed data checklists were removed from the questionnaires after data collection, and codes were given to each questionnaire. The removed checklists were kept in a safe place until discarded.

### Description of important terms and measurement

*Maternity waiting home (MWH)* refers to residential lodging, located near a qualified medical facility, where women in their final weeks (2–3 weeks) of gestation from geographically isolated areas can wait for their birth and be moved to a nearby health facility shortly before childbirth, or earlier should complications arise^3^.

*Knowledge and attitude* participants knowledge about MWH was measured using 7 “Yes” or “No” questions. “Yes” was denoted by “1” and “No” was represented by “0”. Then the sum average for a respondent above the median was considered good knowledge and below the median was considered poor knowledge. Likewise, participants’ attitude towards MWH was measured using 5 points Likert scales (very disagree, disagree, neutral, agree, and very agree). Five questions were used to measure attitude. Average scores above median were considered favorable attitude whereas average scores below median were considered unfavorable attitude^24^.

*Utilization of MWH* refers to pregnant women’s stay at MWH at least for 1 day.

*Couple* refers to a married husband and wife who were living together at the time of this study.

*Male partner* refers to a husband of a woman living with her at time of this study.

*Intervention dummy (Interv*_*i*_*)* is a dummy variable showing control (= 0) and intervention (= 1).

*Post-intervention dummy (Post*_*t*_*)* is a dummy variable indicating pre (= 0) and post (= 1) intervention, i.e., if an outcome happened after intervention (= 1) or otherwise (= 0).

Interaction term (Interv*Post)_*ti*_ is a dummy variable indicating whether the outcome was observed in the intervention group AND it was observed after the intervention (= 1) or any other case (= 0).

### Statistical analysis

The data analysis was performed using STATA version 14.0. The participants were assigned to clusters based on where they lived at the start of the trial. The proportions of participants who had good knowledge about MWH, and had favorable attitude towards MWH were computed at baseline and endline for both intervention and control groups. The outcome measures between the intervention and control arms were compared using the Pearson’s chi-square test of independence. The difference-in-difference (diff-in-diff) estimator was used to estimate the net effect of the intervention, as the baseline outcome did not determine the cluster allocation^35^. To perform the diff-in-diff analysis, we created treatment dummy (*Interv*_*i*_), post dummy (*Post*_*t*_), and the interaction term dummy (Interv*Post)_*it*_, and run the linear regression. The regression coefficient β was used to determine the diff-in-diff estimate and the corresponding confidence intervals were taken. Furthermore, we performed generalized linear model regressions to determine the odds of outcomes between the intervention and control groups. The data were analyzed using an intention-to-treat approach. The statistical significance was declared at p < 0.05 with 95% confidence interval.

### Ethics approval

Ethical approval letter was received from the IRB of Jimma University with reference number JUIRB-33/22, dated 09/02/2022. We received a letter of permission from the Health Department of the Hadiya Zone, Southern Ethiopia. The study participants were informed about the objective of the study, and written informed consent was obtained from each participant prior to the start of data collection. The study was performed in accordance with the national and international guidelines such as Declaration of the Helsinki, and the results were reported as per the CONSORT checklist for cluster randomized trial.

## Results

### Sociodemographic characteristics of the study participants

**Figure 2:**
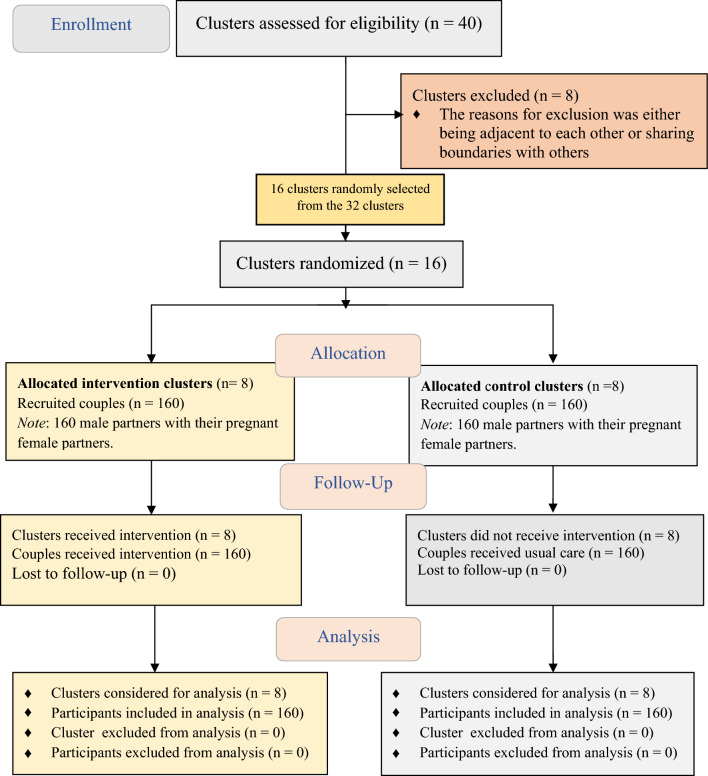
CONSORT flow diagram for a cluster-randomized trial in rural Ethiopia, Sep 15, 2022 to Apr 30, 2023.

Data were collected from 320 couples who participated in the study (160 couples from the intervention group and 160 couples from the control group). There was no loss to follow up (Fig. 2). The data collected from male partners were used in this analysis. Nearly half (45%) of the participants were in the age group (35–49) and the mean age was 38.27 (SD ± 6.42) years. More than half (53.8%) of the participants were from the Hadiya ethnic group and were Protestant religion followers (55% from the intervention and 52.5% from the control groups). Approximately two-thirds (60%) of the participants had no formal education (60% from the intervention and 60% from the control groups). Likewise, two-thirds of the participants were farmers (60% from the intervention and 60% from the control groups). There was no significant differences observed in sociodemographic characteristics between the intervention and control groups (Table 3).

**Table 3:** Sociodemographic characteristics of the participants from a cluster-randomized trial in rural Ethiopia, Sep 15, 2022 to Apr 30, 2023.

Variables	Category	Intervention (n = 160)		Control (n = 160)	
		Frequency	Percent	Frequency	Percent
Age group	20–34	48	30	48	30
	35–49	72	45	72	45
	≥ 50	40	25	40	25
Ethnic group	Hadiya	88	55	84	52.5
	Silte	36	22.5	36	22.5
	Others	36	22.5	40	25
Religion	Protestant	88	55	84	52.5
	Muslim	32	20	32	20
	Others	40	25	44	27.5
Education status	No schooling	96	60	96	60
	Primary education	44	27.5	44	27.5
	Secondary education	20	12.5	20	60.5
Occupation	Farmer	96	60	96	60
	Merchant	40	25	40	25
	Gov’t employee	12	7.5	12	7.5
	Others	12	7.5	12	7.5
^a^Household income/month	Below 7,985 ETB	140	51.5	132	48.5
	≥ 7985 ETB	20	41.7	28	58.3

^a^Monthly HH income cut-off point was taken from Anker Research Institute national living income estimates for rural Ethiopia. The 2021 estimated reference value for rural households in Ethiopia was Birr 7985 per month.

### Effectiveness of the intervention on male partners’ knowledge of and attitude towards MWHs

Health education intervention provided to couples significantly improved male partners’ knowledge of and attitude towards MWHs. Male partners’ knowledge about MWH was increased by 39.4% in the intervention group, while it was increased by 3.8% in the control group. Thus, the net effect (diff-in-diff) of the intervention on increasing good knowledge about MWH was 35.6% (95% CI 31–40.2%, p < 0.001) among male partners. Likewise, the level of favorable attitude towards MWH among male partners’ was increased by 40.6% in the intervention group, whilst it was increased by 4.4% in the control group. As a result, the net effect of the intervention on increasing favorable attitude towards MWHs was 36.2% (95% CI 31.1–41.3%, p < 0.001) (Table 4).

**Table 4:** Statistical summary of the outcome variables from a cluster-randomized trial in rural Ethiopia, Sep 15, 2022 to Apr 30, 2023.

Outcome variables	Category	Baseline (n = 320)			Endline (n = 320)		
		Intervention N (%)	Control N (%)	P- value	Intervention N (%)	Control N (%)	P-value
Knowledge	Good	65(40.6)	61(38.1)	0.37	128 (80)	67 (41.9)	< 0.001
	Poor	95(59.4)	99(61.9)		32 (20)	93 (58.1)	
Attitude	Favorable	56(35)	50(31.2)	0.28	121 (75.6)	57 (35.6)	< 0.001
	Unfavorable	104(65)	110(68.8)		39 (24.4)	103(64.4)	

Moreover, generalized linear model regressions were performed to compare the odds of outcomes between the intervention and control groups both at baseline and endline. At baseline, the odds of having good knowledge about MWH (AOR: 1.11, 95% CI 0.71–1.74, P = 0.46), and favorable attitude towards MWH (AOR: 1.18, 95% CI 0.74–1.88, p = 0.48) did not show statistically significant difference between the groups. However, at the endline, male partners in the intervention group were 5.5 times more likely to have good knowledge about MWHs (AOR: 5.55, 95% CI 3.37–9.14, p < 0.001), and 5.6 times more likely to have favorable attitude towards MWHs (AOR: 5.61, 95% CI 3.45–9.10, p < 0.001) compared to those in the control groups.

## Discussion

This cluster-randomized trial revealed that health education provided to couples significantly improved male partners’ knowledge of and attitude towards MWHs in rural Ethiopia. The couples in the intervention group received health education on the importance of MWHs including paternal support whereas those in the control group received the usual standard care. The health education was provided to the couples at three contact points (first at group health education and then during the two home visits) by the health extension workers who were already working in the health system. The outcome measurements were taken before and after the intervention at 6 months interval.

The male partners’ knowledge about MWH was significantly increased in the intervention group compared to those in the control group. The net effect of the intervention on improving male partners’ knowledge about MWH was 35.6%. Besides, male partners in the intervention group were nearly 6 times more likely to have good knowledge about MWH compared to those in control group. This implies that providing health education to couples can improve male partners’ awareness about the maternal health services including MWHs. In observational studies, male partners’ awareness was mentioned as one of the key factors positively associated with their involvement in maternal health services including antenatal care, health facility delivery, and MWHs^12,36,37^. Initiatives aimed at increasing male partners’ awareness were also recommended to improve their involvement and ensure women’s access to maternal and newborn health services^38–40^. Therefore, creating male partners' awareness through couple education can help them to have a better understanding of MWHs and ease women’s access to maternal and newborn health services, including MWHs.

Furthermore, male partners’ attitude towards MWH was significantly improved in the intervention group as compared to those in the control group. The net effect of the intervention on improving favorable attitudes toward MWH was 36.2% among the male partners. In addition, male partners in the intervention group were nearly 6 times more likely to have favorable attitude towards MWH compared to their counterparts. This indicates that giving health education to couples can improve male partners’ attitude towards maternal health services including MWHs. In literature, male partners attitude was mentioned to be associated with their involvement in maternal health services and women’s access to maternal health services such as antenatal care, health facility birth, postnatal care, and utilization of MWHs^39,40^. Therefore, providing health education to couples can improve male partners attitude towards MWHs and facilitate women’s access to maternal health services including antenatal care, health facility delivery, and postnatal care.

This study had several strengths. The response rate was high with zero dropouts. The data collection tools were adopted from the validated national Ethiopian Demographic Health Survey documents^33^ and global framework for assessing male involvement in maternal health^34^, and pretested. We performed a cluster-randomized trial design with a robust statistical analysis techniques such as GLM and diff-in-diff models to control the possible confounders and estimate net effect on the outcomes. Besides, the intervention was provided by the health extension workers who were already working in health system. Providing the intervention by the health extension workers was vital to ensure the health system ownership and sustainability of the current intervention activities in the future.

This study had also some limitations. First, only outcome assessors were blinded (i.e., the allocation was concealed from the outcome assessors). We could not blind the study participants due to the nature of the study. This might have introduced the social desirability bias in the outcomes. Second, the outcomes were assessed through self-report. This might have introduced a recall bias into the study; though any threats to validity related to the precise estimation of the outcomes distributed equally over control and intervention groups since we randomized the clusters.

## Conclusion

This study revealed that providing health education to couples can improve male partners’ knowledge of and attitude towards MWHs in rural Ethiopia. Policymakers and health system leadership can adopt the current intervention modalities to improve male partners’ knowledge and attitude towards MWHs and other maternal health services in rural Ethiopia.

### Supplementary Information

Supplementary Information 1.

Supplementary Information 2.

Supplementary Information 3.

## Data Availability

The required data is available in the manuscript. Additional data will be made available from the corresponding author up on request.
