# Supplementary material for: Effect of couple-based health education on male-partners knowledge and attitude towards maternity waiting homes in rural Ethiopia: a cluster-randomized trial
Source: Sci Rep. 2023 Oct 27;13:18446. doi: 10.1038/s41598-023-45681-4 (PMC10611718; doi:10.1038/s41598-023-45681-4)
Supplement: Supplementary file 1 — Supplementary Information 1. [file 41598_2023_45681_MOESM1_ESM.pdf]

S1 Appendix: Study Protocol

STUDY PROTOCOL

Title of Research Project

Effectiveness of couple-based health education on utilization of maternity waiting homes in rural Ethiopia: A Cluster-Randomized Controlled Trial

Trial Reg.: ClinicalTrials.gov Identifier: NCT05015023

Investigators and their affiliations:

S.No.	Name	Academic background	Parent institution
1	Teklemariam Ergat Yarinbab	PhD-RH Fellow, MPH Epidemiology	Jimma University, Ethiopia
2	Hailay Abrha Gesesew	PhD Public Health, MPH Epidemiology	Torrens University Australia, Adelaide, Australia
3	Margo Shawn Harrison	MD, MPH, Gyn/Obs specialist	University of Colorado, Denver, CO, US
4	Tefera Belachew	PhD, MSc, MD Professor of Nutrition	Jimma University, Ethiopia

Investigators email addresses:

Teklemariam Yarinbab: teklemariam36@gmail.com

Hailay Gesesew: hailushepi@gmail.com

Margo Harrison: margo.harrison@gmail.com

Tefera Belachew: teferabelachew2@gmail.com

February 2022
Jimma, Ethiopia

Table of contents

List of Tables 3

List of Figures 3

Protocol summary 4

Introduction 5

Methods and materials 6

Objectives 6

General Objective 6

Specific Objectives 6

Study design 6

Study setting 6

Sample size calculations 7

Eligibility criteria 8

Participants' selection 8

Randomization and blinding 9

Information contamination reduction 10

Brief description of the intervention 10

Implementation of the intervention 10

Reporting format [CONSORT Flow Diagram] 16

Usual standard of care 17

Available MWH services 17

Compliance parameter 17

Primary outcome 18

Secondary outcomes 18

Reliability of measures 18

Trial activity schedule 18

Sampling procedure 19

Data collection methods 19

Confidentiality 20

Data management 20

Description of important terms and measurement 20

Statistical analysis 21

Data monitoring.....21
Plan of dissemination.....21
Discussion.....21
Protocol amendment.....21
Declarations.....22
Acknowledgements.....22
Ethics approval.....22
Budget plan for the study.....22
REFERECES.....23

List of Tables

Table 1: Details of intervention activities.....12
Table 2: Summary of the trial protocol.....18
Table 3: Schedule of enrolment, interventions, and assessments.....19
Table 4: Budget plan.....22

List of Figures

Figure 1: CONSORT Flowchart.....16
Figure 2: Sampling Procedure.....20

Protocol summary

Background: The majority of maternal deaths occur in low-resource countries such as Ethiopia. Maternity waiting homes (MWHs) can be used as an approach to improve maternal health outcomes by bringing women living in underserved locations to obstetric facilities. However, the use of MWHs in Ethiopia is low. Therefore, it is important to investigate strategies that can effectively improve MWH use in rural Ethiopia.

Methods: This study will assess the effectiveness of health education provided to couples on the uptake of MWHs among eligible pregnant women in rural Ethiopia. The couples knowledge and attitude towards MWHs will be assessed as well. Cluster-randomized controlled trials will be the study design. The trial will have intervention and control arms. Randomization occurs at the cluster level. Kebeles are clusters. There will be a total of 16 clusters divided into 8 intervention and 8 control clusters. The outcome assessors will be masked. The trial sample size will be 320 couples (160 in each arm). The intervention will be performed for six months. The content of the intervention will be group health education, take-home print health messages, and individual home visits. Health education will be delivered to the intervention group at baseline, and specific leaflets will be provided. Home visits will be conducted two times (i.e., at 3rd and 5th months of the intervention period). The control group will receive the standard care. Baseline and end line data will be collected. An intention to treat approach will be considered. The Chi-square test will be used to assess the statistical difference between the groups, and the difference-in-differences model will be used to measure the effectiveness of the intervention. Furthermore, generalized linear model regressions will be used to estimate the odds of outcomes between the intervention and control groups. The SPSS and/or STATA software will be used to perform the data analysis.

Discussion: The study findings can inform the policymakers and healthcare practitioners to use the intervention modality to improve women's access to maternal health services including MWHs maternity waiting home use among pregnant women with limited access to maternal health services in rural Ethiopia and elsewhere.

Budget: The total estimated cost of the study is 222,768 ETB (equivalent to $4,588 USD).

Trial registration number: ClinicalTrials.gov Identifier: NCT05015023, date of first registration 20/08/2021.

Keywords: maternity waiting home; health education, couples, pregnant women

Introduction

The sustainable development goals target 3.1 aims to reduce the global maternal mortality ratio to less than 70 per 100,000 live births by 2030. [1] Estimates for 2017 show that some 810 women die every day from pregnancy- or childbirth-related complications around the world. The estimate further declared that 295, 000 women died during and following pregnancy and childbirth, globally. Consequently, the global maternal mortality ratio in 2017 is estimated at 211 maternal deaths per 100,000 live births which is still far from the sustainable development goals target. [2] The majority (94%) of this was reported to be occurred in low-resource settings. [3]

Sub-Saharan Africa (SSA) and Southern Asia accounted for approximately 86% (254,000) of the estimated global maternal deaths in 2017. The SSA alone accounted for roughly two-thirds of maternal deaths and the maternal mortality ratio for Ethiopia is estimated to be 401 per 100,000 live births for the same year. [2] This is more than four-fold higher than the global target. Most maternal deaths are preventable.[3] All women need access to high quality care in pregnancy, and during and after childbirth. It is particularly important that all births are attended by skilled health professionals, as timely management and treatment can make the difference between life and death for the mother as well as for the newborn. [2,3]

Delays in accessing health care has been identified as the primary cause of maternal mortality, and this is usually attributed to the long distances that women must travel to reach health facilities. [4] MWHs have been identified as an approach to improve maternal health outcomes by bringing women living in geographically isolated and undeserved areas closer to a healthcare facility that provides emergency obstetric care. [5] MWHs are lodgings located near healthcare facilities where women can await their delivery date and be transferred to health facility shortly before delivery, or earlier should complications arise. [6] MWHs have been implemented in Ethiopia over the last three decades. [7] However, studies have shown that MWH utilization is low in Ethiopia. [8]

The World Health Organization conditionally recommended that the quality of evidence on the utilization of MWHs be poor and insufficiently documented, and further recommends additional research on “what strategies could be effective” in increasing the utilization of MWHs and improving other key maternal health outcomes. [9] Moreover, studies have shown that women’s use

of MWHs largely depends on their husbands' decisions. [10,11] Therefore, this study is designed to evaluate the effectiveness of educating couples on MWH use in rural southern Ethiopia.

Methods and materials

Objectives

General Objective

- To examine the effectiveness of couple-based health education on utilization of MWHs in rural Ethiopia: a cluster randomized controlled trial

Specific Objectives

- To assess the effect of couple-based health education on male-partners knowledge and attitude towards MWHs in rural Ethiopia.
- To assess the effect of couple-based health education on maternal knowledge and attitude towards MWHs in rural Ethiopia.
- To evaluate the effectiveness of couple-based health education in improving utilization of MWHs in rural Ethiopia.

Study design

We will use parallel arms cluster-randomized controlled trial design to examine the effectiveness of couple-based health education improving couples knowledge, attitudes and maternal use of MWHs in rural Ethiopia.

Study setting

The research will be conducted in Hadiya Zone of southern Ethiopia. Hadiya Zone is a second-order administrative division located in Southern Nations Nationalities and Peoples Regional State. It is divided into 4 town administrations and 13 rural districts with a total of 359 clusters or Kebeles' (the smallest administrative units). The livelihood of the population mainly depends on agriculture. There are 4 hospitals, 61 health centers, 317 health posts, and 30 MWHs in the Zone. The total population is around 1.2 million of which 619,170 are women. [12] In 2022, about 60,304 women are estimated to be pregnant. For this study, we will select the districts most appropriate for the intervention based on the availability of the MWHs in the districts. Then clusters and participants will be recruited from those districts.

Sample size calculations

The Hooper and Bourke method for cluster randomization studies of parallel arms with repeated cross-sections is used to calculate the sample size. [13] To illustrate within intracluster correlation coefficient (ICC) and between ICC, the technique comprises the measurement of two design effects, with the product of the two being used to inflate the sample size for individual randomization. The within ICC is the correlation between any two pregnant women in the same cluster, while the between ICC is the correlation between any two pregnant women in different clusters. The first design effect (dc) attributable to cluster randomization is measured using a within ICC of 0.05 obtained from a community-based cluster randomized trial in Ethiopia. [14] The design effect (d_c) is calculated as:

$$\mathrm{dc} = 1 + (m - 1)\rho$$

Where m is the cluster size assumed to be 20 (i.e., the total number of pregnant women who will be questioned in each cluster) and ρ is the within ICC.

The second design effect (d_r) attributable to repeated evaluations (baseline/endline) is calculated by using the within ICC and a cluster autocorrelation coefficient (π) of 0.80. [13]

The second design effect (d_r) is calculated as:

$$d_r = (1 - r^2)$$

Where $r = \left(\frac{m\rho\pi}{d_c}\right)$

The required sample size is then calculated by multiplying the 'sample size assuming individual randomization' by both design effects (dc & dr). It is calculated as:

$$n = \left\lfloor \frac{(a+b)^2 * (p_1q_1 + p_2q_2)}{(|p_1 - p_2|)^2} \right\rfloor * d_c d_r$$

Where:

n represents the sample size in each of the arms i.e., intervention and control

a represents conventional multiplier (1.96) for alpha $(\alpha = 0.05)$ and b represents conventional multiplier (0.842) for power $(1 - \beta = 0.80)$

p_1 represents proportion of post-intervention users of MWH and q_1 represents proportion of post-intervention non-users of MWH.

p_2 represents proportion (50%) of users of MWH taken from a study in Gurage Zone, Southwest Ethiopia [15] and q_2 represents pre-intervention proportion of non-users of MWH.

$|p_1 - p_2|$ *an effect size* - is an absolute change in proportion of MWH utilization after intervention. It is estimated to be 20%.

In addition, the following parameters are considered: 95% CI, 80% power, 1:1 allocation ratio of intervention to control, 10% potential loss to follow up, and tabulated sample size $(n_0 = 199)$ required to detect a difference in two proportions at 5% significance level with 80% power in literature. [16] According to Hooper and Bourke, the number of clusters (K) for the sample is determined using the formula $K = (n_0 d_c d_r)/m$. The final sample size is calculated by substituting the specified values into the above formula. Hence, a total of 16 clusters are needed, with an approximated final sample size of 320 couples. The two arms each will have 160 couples (pregnant women with their male partners).

Eligibility criteria

The study participants will be couples living together where the women are pregnant in the beginning of second trimesters of pregnancy (14-16 weeks of gestation) and had given birth in the last 5 years before the current pregnancy, permanent residents of the study area, living ≥2 hours of walking distance from the nearest health facility and/or have no or limited access to public transportation, and are willing to participate in the study.

Participants' selection

Hadiya Zone was one of lowest performers in SNNPR regarding MWH use in 2021. This situation drew the attention of the researchers to perform this study in this area. First, health centers with functional MWHs will be identified based on the MWH functionality criteria adopted from national guideline. During the assessment; the availability of staff, beds for sleeping, water supply, kitchen and cooking utensils, bathrooms, and latrines will be considered as crucial to declare the functionality of MWHs.

In Ethiopian health system, one health center serves an estimated 25,000 population with a catchment of five Kebeles (clusters). Each Kebele contributes 5000 population. Secondly, all the Kebeles under each health center catchments will be listed. Thirdly, all non-adjacent Kebeles relatively from the health centers will be identified. Next, 16 non-adjacent Kebeles will be chosen. At least one cluster (Kebele) will be left between non-adjacent Kebeles, and this will serve as a buffer zone.

The selected 16 non-adjacent Kebeles will be randomly assigned to intervention and control groups using simple randomization. Census and/or health post records will be used to identify pregnant women. Then, women will be asked their Last Menstrual Period (LMP) to estimate the gestational age. Women who are in the beginning of second trimesters (14 -16 weeks of gestation) will be listed. This list will be used as a sampling frame. The study participants will be selected from each Kebele using simple random sampling technique.

Participants who consent to involve in the study with their male partners (husbands) will be included in the study. Women and their male partners will be requested to sign an informed consent to ensure voluntary participation. Once consent is obtained, each participant will be interviewed to complete a baseline survey. The baseline questionnaires contain socio-demographic and obstetrics characteristics, health services including MWH use, paternal and maternal knowledge and attitude, and male partner involvement.

Randomization and blinding

Kebeles' will be the randomization unit whereas the observation or analysis units will be the individual study participants. The selected 16 non-adjacent Kebeles will be listed alphabetically. A restricted randomization with a 1:1 allocation will be used to assign the Kebeles to the intervention or control groups. In Microsoft Excel 2010, a list of random numbers will be created, and the generated values will be fixed by copying them as "values" next to the alphabetic list of the Kebeles. The first eight will be chosen as intervention clusters, and the last eight will be chosen as control clusters, in ascending order based on the produced random numbers. Moreover, a statistician who is blind to the study groups and is not involved in the research will create the allocation sequence and randomize the clusters. The allocation of Kebeles to the intervention or control groups will be hidden from the data collectors or outcome assessors.

Information contamination reduction

The study participants in the control group may be informed about the intervention in various social occasions such as weeding, marketing, or funeral ceremony. This may affect intervention outcome unfavorably. Therefore, leaving at least one cluster (Kebele) between non-adjacent clusters may reduce such contamination.

Brief description of the intervention

The intervention has three components: group health education, provision of take-home print health messages, and home visits. The study participants in the intervention group will receive the intervention alongside the usual care. The couples will receive the intervention together. The intervention will be provided at three contact points. The first contact will be the group health education at baseline whereas the second, and third contacts will be home visits. The control group will receive the usual care.

Implementation of the intervention

The intervention will be provided to the couples in the intervention group. Group health education, provision of leaflets (print health messages), and home visits will be conducted. Health education will be provided once in the first month whereas the home visits will be performed two times at two months intervals (i.e., at 3rd and 5th months). Print health messages will be provided three times (i.e., at first contact during health education and at each of the two home visit).

The health education session will be provided in a group for 90-120 minutes. All participants in the intervention within a Kebele will be gathered at one common place and receive health education. Health extension workers and Kebele leaders will select the place of health education and invite the participants. Both women and their male partners will be invited to receive the health education. This will be done in all eight intervention Kebeles, and as a result, eight health education sessions will be performed at baseline.

Health education will address the importance and kinds of paternal support, and purpose and benefits of staying in MWHs. The services available at MWHs, the benefits of staying at MWHs, the right time to visit MWHs, and the importance of paternal support will be discussed. Types of paternal support during pregnancy and MWH stay, such as allowing a spouse to stay at MWH, accompanying her to MWH, providing financial support during MWH stays, providing food and other necessary materials, looking after the home, and caring for the remaining children at home,

will be addressed. Health messages in leaflets focus on paternal support and the purpose and advantages of MWHs. Next, the schedule of home visits will be proposed through discussions with participants, with an emphasis that both women and their male partners will be contacted at their residence.

The first home visit will be conducted two months after the health education intervention (i.e., in the 3rd month of the intervention). During this visit, both women and their male partners will receive advice regarding antenatal care, paternal support, and MWHs. Male partners will be advised on how to support their wives and encourage them to use antenatal care, stay at MWHs, and deliver at a health facility. The couples will be asked to discuss their perceptions of antenatal care, MWH use, and health facility delivery. Based on their perceptions and actual observed practices, they will be advised. Leaflets with the same health messages at baseline health education will be provided. Repeated visits will be made if the couple is absent.

The second home visit will be conducted two months after the first home visit (i.e., in the 5th month of the intervention). During this visit, paternal support, birth preparedness plans, and intention to use MWH will be assessed. Leaflets containing messages regarding possible risks of home delivery and advantages of staying at MWHs and institutional delivery will be provided. Any misunderstandings regarding MWHs will be clarified through discussion. Male partners will be advised to continue supporting their wives. The expected delivery date will be estimated and a possible appointment to arrive at the MWH will be made. To make the woman and her partner remember the appointment, a written invitation letter will be provided. However, the woman will be advised to attend antenatal care, follow the advice of health professionals, and come to a health facility if she feels any illness (Table 1).

Table 1: Details of intervention activities

Intervention packages	List of activities	Duration	Responsible body
Group health education	Focus of health education<br>- Purposes and benefits of staying in MWHs<br>- Explain what MWH is<br>- Purposes of MWH<br>- Types of services available at MWHs<br>- Benefits of staying at MWHs<br>- Who should use MWHs<br>- When to go to MWH<br>- Benefits and kinds of paternal support<br>- Define what does paternal support mean in the current context (*Hint: In this particular context paternal support refers to any kind of physical, material, or emotional support a husband provides to his expectant wife*.)<br>- Emphasis on paternal support related to MWHs and discuss types of paternal support such as allowing a spouse to stay at MWH, accompanying her to MWH, providing financial support during MWH stays, providing food and other necessary materials, looking after the home, and caring for the remaining children at home etc.<br>- Provide leaflet to each couple at the end of the health education sessions<br>- The health messages in the leaflets will focus on paternal support, and the purpose and advantages of MWHs.	90-120 minutes per group	Trained Health Extension Workers

First home visit	During first home visit:<br>- Assess the couples practice regarding antenatal care and paternal support<br>- Advise the MPs on how to support their wives during pregnancy<br>- Tell MPs to encourage their wives to use antenatal care<br>- Further, ask the couples to discuss their perceptions of antenatal care and paternal support and MWH use<br>- Advise them based on their perceptions and actual observed practices<br>- Provide them leaflets (same leaflet provided at baseline)	30-45 minutes per couple	Health Extension Workers
Second home visit	During second home visit:<br>- Assess the practice of paternal support and couples intension related to MWH use<br>- Assess any birth preparedness plans including readiness to use MWHs, financial preparations, transportation arrangements to MWH, and choice of place of delivery.<br>- Discuss the possible risks of home delivery and advantages of staying at MWHs and institutional delivery<br>- Clarify any misunderstandings regarding MWHs through discussion<br>- Advise the MPs to continue supporting their expectant wives<br>- Estimate the expected delivery date and make possible appointment to go to the MWH<br>- Provide them a written invitation to MWH	30-45 minutes per couple	Health Extension Workers

Sample Leaflet (Print health message)

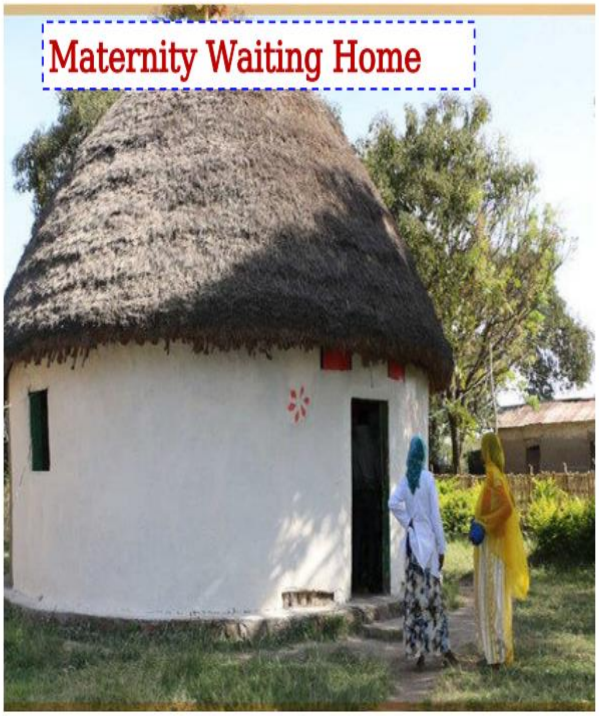

Have you ever heard about maternity waiting home?

Maternity waiting home – is a shelter where pregnant women in last weeks of gestation can await their delivery and be transferred to a nearby medical facility shortly before delivery, or earlier should complications arise.

When should woman stay at maternity waiting home?

- Pregnant woman stays at maternity waiting home in last weeks of pregnancy.
- Health care professionals can give a woman information about when to go to maternity waiting home.

Benefits of Maternity Waiting Home

- To avoid maternal problems caused by travelling long distances during labour
- To receive close medical follow-up in last weeks of pregnancy
- To stay near health facility and receive medical care during labour and delivery
- To be easily transferred to health facility for labour and delivery, or should complications arise
- It is Safe and comfortable place for pregnant woman to stay before delivery
- It allows woman to network with others and learn how to care for herself and the baby

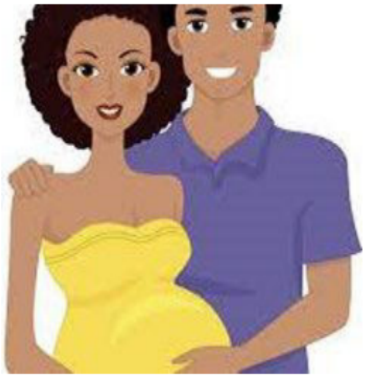

Can male partners (husbands) involvement improve maternal health?

Male partners involvement during pregnancy and child birth can improve maternal health outcomes.

What does male partners involvement mean?

Male partners involvement refers to husbands engagement in maternal health issues. Maternal health can not be ensured without male partners participation.

- ⇒ Do you support your wife?
- ⇒ What kinds of support do you provide to your pregnant wife?

Male partner's (husband's) support is essential during pregnancy and childbirth.

Kinds of support a husband provide to his pregnant wife are:

⇒ **Social support**

Example: Taking care of children, household care and/or other work

⇒ **Financial support**

Example: cover costs for transportation to health facility, food & medications

⇒ **Emotional support**

Example: Exercise free couple-communication, accompany his wife when she goes to health facility for medical care, encourage her to be strong while she feels discomfort, and/or decide to use health facility for delivery

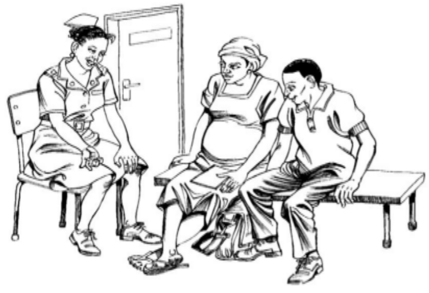

A woman attending ANC with her husband

Tips for husbands

What to do?

- ⇒ Communicate with your wife freely about reproductive health matters
- ⇒ Support your wife by sharing household responsibilities such as taking care of children
- ⇒ Cover transportation and medication costs when she visit health facility
- ⇒ Accompany her when she goes to health facility for ANC visit
- ⇒ Show her sense of empathy when she feels any discomfort
- ⇒ Do plan of maternity waiting home use with your wife
- ⇒ Do plan of labour and delivery with your wife

Contact

Teklemariam Ergat Yarinbab (MPH)
Assistant Professor of Epidemiology
Email: teklemariam36@gmail.com
Cell phone: +251930504891

February 2022
Jimma University

Reporting format [CONSORT Flow Diagram]

The study findings will be reported using Consolidated Standards of Reporting Trials (CONSORT) for cluster randomized trials criteria [17] (Figure 1).

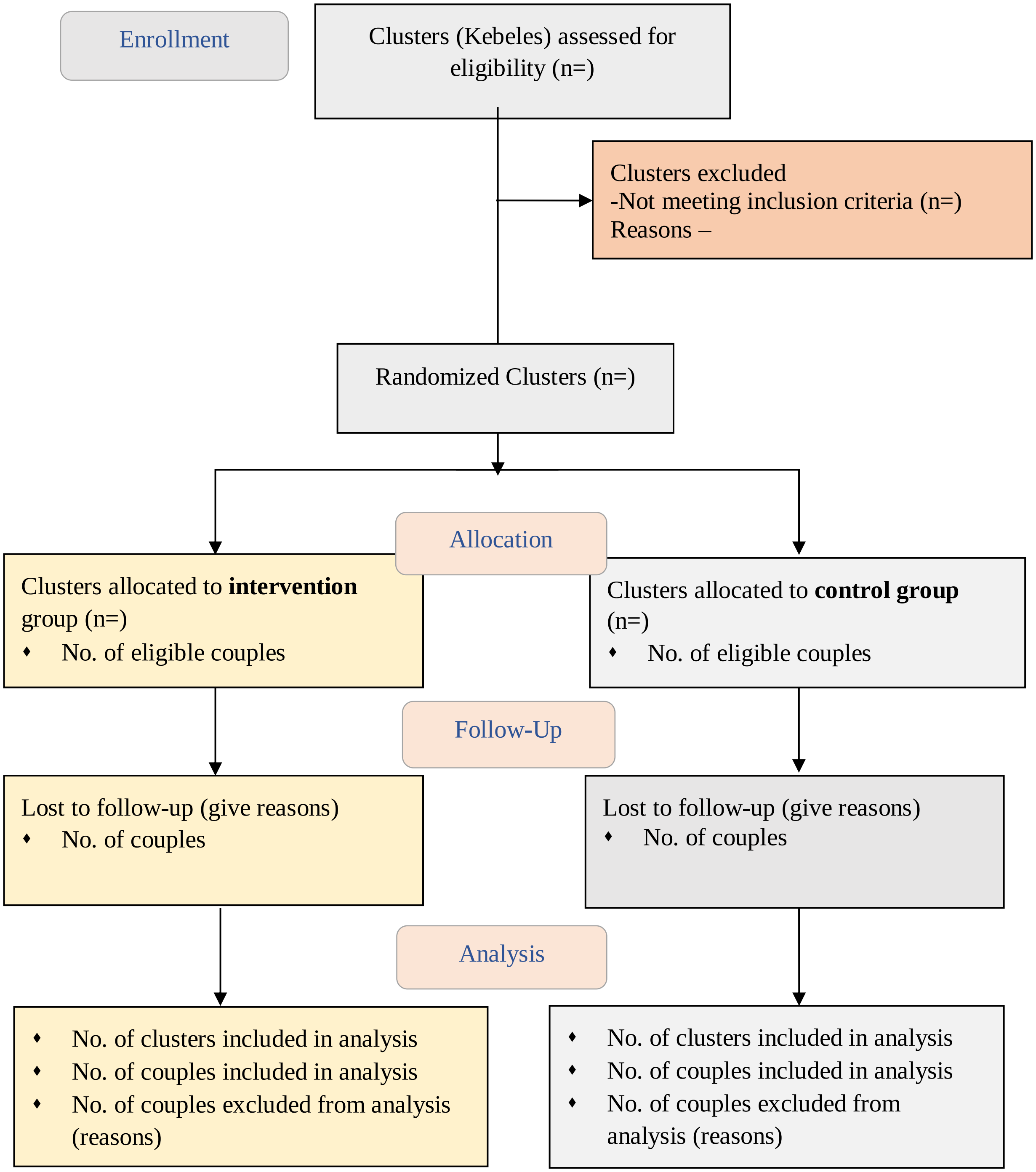

Figure 1: CONSORT Flowchart

Usual standard of care

Preventive and promotive health services are provided to the community in the study setting through rural health extension programs. The rural health extension program packages include disease prevention and control, family health, personal hygiene, environmental sanitation, health education, and communication. [18] Consequently, under the family health package, rural health extension workers promote contraceptive use, antenatal care, institutional birth, and other family health issues. These services are common to all households in the study setting. Other than these usual services, the control group will not receive the basic components of current intervention, such as group health education and home visits, which aim to educate and counsel women and their male partners together regarding paternal support and MWH use. The control group will receive the current intervention after the completion of the study through routine rural health extension programs.

Available MWH services

Women who stay at MWHs will receive the services based on the national guideline. [19] The women will be registered and provided sleeping rooms at their arrival. The antenatal check-ups and follow up by health professionals are the basic available health services. The MWH staff will be informed to welcome the male partners of the pregnant women and encourage them to support their spouses during their stay at the MWHs. In addition to the registration book at the MWH, a separate registration format will be prepared for this study purpose.

Compliance parameter

The number of participants who attended health education at baseline and contacted at the two home visits will be used to determine the participants' compliance with the intervention. We will use attendance sheets to determine participants' compliance with the proposed intervention packages. However, for various reasons, the participants in the intervention may not comply with the intervention. This may have undesirable effects on women's use of MWHs. This will be considered a dose–response function during the data analysis (Table 2).

Table 2: Summary of the trial protocol

Content of intervention	Dosage	Frequency	Duration	Compliance parameter
Group health education at baseline and leaflets	90-120 minutes	Once	One month	Number of participants attended the health education sessions
Home visits and leaflets	30-45 minutes	Twice	Two months	Number of participants contacted at home visits

Primary outcome

The proportion of MWH use among participants will be measured. The use of MWH can be traced through individual surveys and MWH record reviews. The proportion of MWH use will be calculated for each trial arm at baseline and end line. The effectiveness of the intervention will be measured as a difference-in-differences in the proportion of MWH use between the trial arms.

Secondary outcomes

The couples knowledge and attitude will be measured at the baseline and endline (after the follow-up is completed). Consequently, the effect of the intervention on the participants' knowledge and attitudes will be compared between the trial arms.

Reliability of measures

The data collection tool will be adapted from different studies such as Ethiopian Demographic Health Survey Documents, global framework for assessing male partners' involvement in maternal health and other relevant sources. The adapted tools will be structured to fit the study context, which contributes to the reliability of the measures. In addition, the reliability of the measurement items will be assessed using Cronbach's alpha and appropriate corrections will be made based on the test findings. The Cronbach's alpha cut-off value will be ≥ 0.70. The data collectors will be trained on the standards and techniques of data collection.

Trial activity schedule

The eligibility screening, obtaining informed consent, participant allocation, and baseline data collection will be performed in the months of September and October 2022. The actual intervention will be performed from November 2022 to April 2023. Then the endline data collection will be conducted two-weeks after the completion of the intervention, i.e., after May 15, 2023.

Accordingly, the participants' enrollment, intervention, and assessment schedule is summarized as follows (Table 3).

Table 3: Schedule of enrolment, interventions, and assessments

			**STUDY PERIOD**						
		**Enrolment & Allocation**	**Post-allocation (Intervention Phase)**						**Close out**
**Time point**		$t_{(-1)}$	t_1	t_2	t_3	t_4	t_5	t_6	$t_{(+1)}$
**Enrolment**	Eligibility screen								
	Informed consent								
	Allocation								
**Interventions**	Health Education + Leaflets								
	Home Visits + Leaflets								
**Assessments**	Baseline data								
	Knowledge & Attitude								
	MWH utilization								

Sampling procedure

Sixteen clusters will be selected based on MWH availability in the area. These clusters will be randomly assigned to intervention and control groups. Census and/or health post records will be used to identify pregnant women in the chosen clusters, resulting in the formation of a sampling frame. The study participants will then be selected using a simple random sampling technique from the sampling frame. Each cluster will have 20 couples (20 wife-husband pairs). The same sample of participants assessed at baseline will be assessed at end line to measure the outcome (Figure 2).

Data collection methods

Pretested structured questionnaires will be used to collect data through face-to-face interviews. Baseline data will be collected at the beginning and the end line data will be collected at the end of the intervention by home-to-home visits of each study participant. Study variables such as socio-demographic characteristics, maternal health service-related variables, male-partners involvement, maternal and paternal knowledge and attitude, and utilization of MWH use will be assessed.

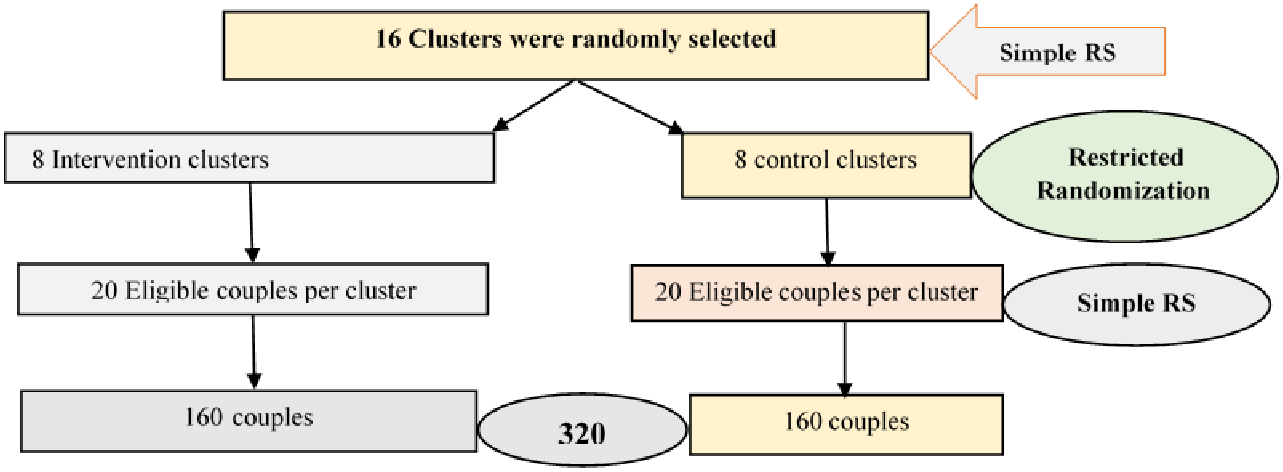

Figure 2: Sampling Procedure

Confidentiality

The confidentiality of study participants will be considered. The completed data checklist will be removed from the questionnaires after data collection, and codes will be given to each questionnaire. The removed checklists will be kept in a safe place until it will be discarded. The codes assigned to each participant will be used.

Data management

Clusters and participant codes will be assigned to each of the completed questionnaires. Data will be entered into Epi info version 7.2.5., edited, and transported to SPSS or STATA software for statistical analysis.

Description of important terms and measurement

Maternity waiting home (MWH)*:* refers to residential lodging, located near a qualified medical facility, where women in their final weeks (2 – 3 weeks) of gestation from geographically isolated areas can wait for their birth and be moved to a nearby health facility shortly before childbirth, or earlier should complications arise. [6]

Knowledge and attitude*:* Participants knowledge about MWH will be measured using 7 “Yes” or “No” questions. “Yes” will be denoted by “1” and “No” will be represented by “0”. Then the sum average for a respondent above the median will be considered good knowledge and below the median will be considered poor knowledge. In addition, participants’ attitude towards MWH will be measured using 5 points Likert scales (very disagree, disagree, neutral, agree, and very agree). Five questions will be used to measure attitude. Average scores above median will be considered favorable attitude whereas average scores below median will be considered unfavorable attitude.

Utilization of MWH: refers to pregnant women's stay at MWH at least for a day.

Couple: refers to a married husband and wife who are living together at the time of this study.

Male partner (MP): refers to a husband of a woman living with her at time of this study.

Statistical analysis

The data analysis will be performed using STATA or SPSS software. The outcome variables will computed at baseline and endline for both intervention and control groups. The outcome measures between the intervention and control arms will be compared using the Pearson's chi-square test of independence. The difference-in-difference (diff-in-diff) estimator will be used to estimate the effect of the intervention. Furthermore, we will perform generalized linear model regressions to determine the odds of outcomes between the intervention and control groups. The data will be analyzed using an intention-to-treat approach. The statistical significance will be declared at p-value < 0.05 with 95% confidence interval.

Data monitoring

Team of field supervisors will be responsible for monitoring and auditing the data during baseline, intervention, and end line assessments.

Plan of dissemination

The trial findings will be communicated to relevant stakeholders in the local community and government bodies. It will also be disseminated to scientific communities through publication of results in reputable international journals.

Discussion

In Ethiopia, the utilization of MWHs is low and the rates vary across regions in the country. Although the WHO recommends further research to investigate strategies that could be effective in increasing the utilization of existing MWHs, interventional research aimed at improving MWH use is scarce in the country. Therefore, this trial aimed to evaluate educating couples as an intervention strategy to improve MWH utilization. The results of this trial may help policymakers and healthcare practitioners to design viable strategies to improve women's access to MWHs.

Protocol amendment

Any modifications to this study protocol, including changes in study objectives, study design, study population, sample size, or study procedures, will be communicated to the Institutional Review Board (IRB) of Jimma University and approved before implementation.

Declarations

Acknowledgements

We thank Jimma University, Institute of Health, for granting ethical approval for this study.

Ethics approval

Ethical approval letter was received from the IRB of Jimma University with reference number JUIRB-33/22, dated 09/02/2022. Consequently, a letter of permission will be obtained from the Health Department of the Hadiya Zone, southern Ethiopia. The study participants will be informed about the objective of the study, and written informed consent will be obtained from each cluster representatives as well as participants prior to the start of data collection.

Budget plan for the study

The total estimated cost of the study is 222,768 ETB (equivalent to $4,588 USD). Jimma University will cover the baseline data collection by providing 60,000 ETB. The rest cost of the study will be covered by the investigators themselves. The investigators will be searching for external donors to cover the study expenses (Table 4).

Table 4: Budget plan

S. No	Budget Category		Participants*#days* #payment/day	Quantity	Unit price	Total cost [ETB]
1	Qualitative data collection	Data collectors	2x5x500			5,000
		Voice recorder		2	3500	7,000
2	Intervention	Baseline	Data collectors = 6x15x500<br>Health educators = 16x300<br>Refreshment = Coffee 10kg*120 + Kolo15kg*100			52,500
		Home visits	○ Visits = 16x15x300			72,000
		Print materials	○ Leaflets = 160x4x0.5			320
		Endline	○ Data collection = 6x15x500			45,000
3	Supervision	Supervisors	Per diem = 2x20x600			24,000
4	Transportation	Fuel (200Litres)	○ Cost = 6348			6,348
5	Materials	Paper		10packs	575	5,750
		Pen/Pencil		2packs	575	1,150
		Ruler		10pcs	120	1,200
		Binder		10pcs	250	2,500
	Grand Total (ET Birr)					222,768

REFERECES

1. World Health Organization. Maternal heath: fact sheet on Sustainable Development Goals (SDGs): health targets [Internet]. WHO; 2017 [cited 2022 Jun 8]. Available from: https://apps.who.int/iris/handle/10665/340843
2. World Health Orgnization. Trends in maternal mortality: 2000 to 2017: estimates by WHO, UNICEF, UNFPA, World Bank Group and the United Nations Population Division: executive summary [Internet]. WHO; 2019 [cited 2022 Jul 5]. Available from: https://apps.who.int/iris/handle/10665/327596
3. World Health Organization. An evidence brief on maternal mortality [Internet]. WHO; 2019 [cited 2022 Jun 18]. Available from: ttps://apps.who.int/iris/bitstream/handle/10665/329886/WHO-RHR-19.20-eng.pdf
4. Singh K, Speizer IS, Kim ET, Lemani C, Tang JH, Phoya A. Evaluation of a maternity waiting home and community education program in two districts of Malawi. BMC Pregnancy Childbirth. 2018;18(1):1-14.
5. Perosky JE, Munro-Kramer ML, Lockhart N, Musonda GK, Naggayi A, Lori JR. Maternity waiting homes as an intervention to increase facility delivery in rural Zambia. Int J Gynecol Obstet. 2019;146(2):266-7.
6. World Health Organization. Maternity waiting homes: a review of experiences [Internet]. WHO; 1996 [cited 2022 Jul 16]. Available from: https://apps.who.int/iris/handle/10665/63432
7. Gaym A, Pearson L, Soe KWW. Maternity waiting homes in Ethiopia: three decades experience. Ethiop Med J. 2012;50(3):209-19.
8. Kassa BG, Ayele AD, Belay HG, Mihiretie GN, Worke MD. Utilisation of maternity waiting homes and its associated factors in Ethiopia: Systematic reviews and meta-analysis. Clin Epidemiol Glob Heal. 2021;12.
9. World Health Organization. WHO recommendations on health promotion interventions for maternal and newborn health 2015 [Internet]. WHO; 2015 [cited 2022 Jul 15]. Available from: https://www.who.int/publications/i/item/9789241508742
10. Sialubanje C, Massar K, Van Der Pijl MSG, Kirch EM, Hamer DH, Ruiter RAC. Improving access to skilled facility-based delivery services: women's beliefs on facilitators and barriers to the utilisation of maternity waiting homes in rural Zambia. Reprod Health.

2015;12(1):1-13.

11. Gurara MK, Geertruyden J-P, Jacquemyn Y, Draulans V. Stakeholders' perspectives on the implementation of maternity waiting homes in rural Ethiopia: a qualitative study. Res Sq. 2021:1-25. https://doi.org/10.21203/rs.3.rs-971096/v1
12. Central Statistical Agency of Ethiopia. Population and housing census 2007 report [Internet]. CSA Ethiopia; 2007 [cited 2022 Jul 8]. Available from: https://catalog.ihsn.org/index.php/catalog/3583
13. Hooper R, Bourke L. Cluster randomised trials with repeated cross sections: alternatives to parallel group designs. BMJ. 2015;350.
14. Alemayehu, M., Medhanyie, A.A., Reed, E. et al. Use of community-based interventions to promote family planning use among pastoralist women in Ethiopia: cluster randomized controlled trial. BMC Women's Health 21, 305 (2021).
15. Getachew B, Liabsuetrakul T, Gebrehiwot Y. Association of maternity waiting home utilization with women's perceived geographic barriers and delivery complications in Ethiopia. Int J Health Plann Manage. 2020;35(1):e96-e107.
16. Campbell MJ, Julious SA, Altman DG. Estimating sample sizes for binary, ordered categorical, and continuous outcomes in 2 group comparisons. BMJ. 1995;311(7013):1145.
17. Campbell MK, Elbourne DR, Altman DG. CONSORT statement: extension to cluster randomised trials. Br Med J. 2004;328(7441):702-708.
18. Wang H, Tesfaye R, Ramana GN V, Chekagn CT. Ethiopia health extension program: an institutionalized community approach for universal health coverage. World Bank Group; 2016 [cited 2022 Aug 18]. Available from: https://openknowledge.worldbank.org/handle/10986/24119
19. FMOH Ministry of Health Ethiopia. Guideline for the establishment of standardized maternity waiting homes at health centres/facilities. Addis Ababa; 2015.
