# Supplementary material for: Effect of couple-based health education on male-partners knowledge and attitude towards maternity waiting homes in rural Ethiopia: a cluster-randomized trial
Source: Sci Rep. 2023 Oct 27;13:18446. doi: 10.1038/s41598-023-45681-4 (PMC10611718; doi:10.1038/s41598-023-45681-4)
Supplement: Supplementary file 2 — Supplementary Information 2. [file 41598_2023_45681_MOESM2_ESM.pdf]

S2 Appendix: MWH Functionality Assessment Checklist

S.No.	Items	Available	Not Available
**1**	Registration books		
**2**	MWH staff (clinical & non-clinical)		
**3**	Beds (staying rooms for women)		
**4**	Electricity (at least power for light)		
**5**	Water supply		
**6**	Television/Radio or any entertainment service		
**7**	Kitchen/designated cooking area		
**8**	Cooking utensils		
**9**	Bathrooms		
**10**	Latrine		
