# Supplementary material for: Effect of couple-based health education on male-partners knowledge and attitude towards maternity waiting homes in rural Ethiopia: a cluster-randomized trial
Source: Sci Rep. 2023 Oct 27;13:18446. doi: 10.1038/s41598-023-45681-4 (PMC10611718; doi:10.1038/s41598-023-45681-4)
Supplement: Supplementary file 3 — Supplementary Information 3. [file 41598_2023_45681_MOESM3_ESM.pdf]

S3 Appendix: Questionnaires (English Version)

Consent Form

Information

Dear/Sr/Madam _____

My name is ___________________________________.

I am working with a research team from Jimma University. We are conducting research entitled "*Effectiveness of educating couples on MWH utilization*". The purpose of the study is to benefit the women living in remote and rural areas. The finding will help those women living in the geographically isolated areas who are suffering from problems of access to health facilities during pregnancy and childbirth. You and your husband are warmly invited to participate in this study. Your contributions will be invaluable to the effectiveness of this research project. You can withdraw after you join the research project at any time.

The information we obtain will be used for the research purpose only. We will not disclose your name in the research report. Any information regarding the research will not be linked to your name. Codes will be given for all the questionnaires for any data consumption.

Consent of the participant

We, I and my wife, understood the objectives of the research and are willing to participate in the study. We put our signatures/finger prints below as confirmation of our consent to participate in this study.

Signatures/finger prints

Husband ______________Date _________

Wife ____________ Date ____________

Section 1: Baseline survey

Woreda: __________ Kebele: _________ Village Name: __________ House No: _____________ Participant Code: ________

Part I: Sociodemographic characteristics

S.No.	Questions	Response
Q101	How old are you?	__________ years
Q102	What is your ethnic group?	1. Hadiya<br>2. Wolyta<br>3. Silte<br>4. Gurage<br>5. Others(specify) _
Q103	What is your religion?	1. Protestant<br>2. Orthodox<br>3. Muslim<br>4. Other _______
Q104	What is your level of education?	1. No formal education<br>2. Grade 1 – 8<br>3. Grade 9 – 12<br>4. College/University
Q105	What is your occupation?	1. Farmer<br>2. Merchant<br>3. Government employee<br>4. Student<br>5. Other ____
Q106	How much do you earn in a month? (household income)	_ _________ Birr/Month [Specify annual income if not feasible]
Q106	How many children do you have?	____

Part II: Male partners’ knowledge about MWH

S.No.	Questions	Hint	Yes	No
Q201	Do you know what MWH is?<br>[If he say yes, ask him to describe what MWH is]	MWH is a residence located in/near health facility where pregnant women stay until childbirth.		
Q202	Do you know the advantages of MWH?<br>[If he say yes, ask him to list at least one advantage]	Example of advantages of MWH: - solves long distance problems, improves mother and newborn health outcomes, and helps pregnant women to get timely medical care.		
Q203	Do you know a health facility that provide MWH services?<br>[If he say yes, ask him where or which health facility]	Place or name of the health facility he knows that has a MWH service.		
Q204	Do you know when a pregnant woman should go to MWH?<br>[If he say yes, ask him when?]	Pregnant women are recommended to go to MWH when they are in last weeks (2-3 weeks) of pregnancy, however this may differ. But at least he should say when she is near her delivery date.		
Q205	Do you know type of health services provided at MWH?<br>[If he say yes, ask him to tell at least one service]	He should respond at least one service, examples: antenatal check-up, follow-up, health education, providing sleeping room services including meals		

Q206	Do you know what husbands [male partners] should do during their spouse stay at MWH? [If yes, ask him to tell you at least two things husbands should do]	Examples: - allowing her to stay at MWH, accompanying her to MWH, providing financial support during MWH stays, providing food and other necessary materials, looking after the home, and caring for the remaining children at home.		
Q207	Do you know what kind of pregnant women are highly recommended to stay at MWHs? [If he say yes, ask him to tell what kind – he should say at least one]	Example: - Pregnant women living in rural & remote areas, have limited access to transportation, women at high risk, have history of pregnancy related complications, etc.		

Part III: Male partners' attitude towards MWH

A five points Likert scale is used to measure male-partners attitude. The numbers are labeled as follows: (1=strongly disagree) (2=disagree) (3=neutral) (4=agree) (5=strongly disagree)

**S.No.**	**Attitude Statements**	**Scale**				
		**1**	**2**	**3**	**4**	**5**
Q301	MWH prevents distance barriers to obstetric care.	1	2	3	4	5
Q302	MWH enables pregnant women to receive obstetric services timely during labor and delivery.	1	2	3	4	5
Q303	Staying at MWH improves mother and newborn health outcomes.	1	2	3	4	5
Q304	Husbands should allow their spouses to stay at MWH.	1	2	3	4	5
Q305	Husbands should provide all necessary support their wives during their stay at MWH	1	2	3	4	5

Section 2: Endline survey

Woreda: __________ Kebele: _________ Village Name: __________ House No: _____________ Participant Code: ________

Part I: Male partners' knowledge about MWH

S.No.	Questions	Hint	Yes	No
Q201	Do you know what MWH is?<br>[If he say yes, ask him to describe what MWH is]	MWH is a residence located in/near health facility where pregnant women stay until childbirth.		
Q202	Do you know the advantages of MWH?<br>[If he say yes, ask him to list at least one advantage]	Example of advantages of MWH: - solves long distance problems, improves mother and newborn health outcomes, and helps pregnant women to get timely medical care.		
Q203	Do you know a health facility that provide MWH services?<br>[If he say yes, ask him where or which health facility]	Place or name of the health facility he knows that has a MWH service.		
Q204	Do you know when a pregnant woman should go to MWH?<br>[If he say yes, ask him when?]	Pregnant women are recommended to go to MWH when they are in last weeks (2-3 weeks) of pregnancy, however this may differ. But at least he should say when she is near her delivery date.		
Q205	Do you know type of health services provided at MWH?<br>[If he say yes, ask him to tell at least one service]	He should respond at least one service, examples: antenatal check-up, follow-up, health education, providing sleeping room services including meals		

Q206	Do you know what husbands [male partners] should do during their spouse stay at MWH?<br>[If yes, ask him to tell you at least two things husbands should do]	Examples: - allowing her to stay at MWH, accompanying her to MWH, providing financial support during MWH stays, providing food and other necessary materials, looking after the home, and caring for the remaining children at home.		
Q207	Do you know what kind of pregnant women are highly recommended to stay at MWHs?<br>[If he say yes, ask him to tell what kind – he should say at least one]	Example: - Pregnant women living in rural & remote areas, have limited access to transportation, women at high risk, have history of pregnancy related complications, etc.		

Part II: Male partners' attitude towards MWH

A five points Likert scale is used to measure male-partners attitude. The numbers are labeled as follows: (1=strongly disagree) (2=disagree) (3=neutral) (4=agree) (5=strongly disagree)

**S.No.**	**Attitude Statements**	**Scale**				
		**1**	**2**	**3**	**4**	**5**
Q301	MWH solves long distance problems for pregnant women.	1	2	3	4	5
Q302	MWH enables pregnant women to receive health services timely during labor and delivery.	1	2	3	4	5
Q303	Staying at MWH improves mother and newborn health outcomes.	1	2	3	4	5
Q304	Husbands should allow their spouses to stay at MWH.	1	2	3	4	5
Q305	Husbands should provide all necessary support their wives during their stay at MWH	1	2	3	4	5
